# Decoding pulmonary infections through targeted next-generation sequencing: comprehensive pathogen profiling in 999 clinical cases

**DOI:** 10.3389/fcimb.2026.1772169

**Published:** 2026-05-15

**Authors:** Haiqiang Hu, Lu Liu, Shuli Yu, Zhenping Liu, Longying Zhu, Chunyan Qian

**Affiliations:** 1 The First People’s Hospital of Linping District, Department of Clinical diagnostics laboratory, Hangzhou, Zhejiang, China; 2 Department of Infectious Disease, Melbourne Medical School, The Peter Doherty Institute for Infection and Immunity, University of Melbourne, Melbourne, VIC, Australia

**Keywords:** mixed infection, pathogen detection, pulmonary infection, respiratory microbiology, targeted next-generation sequencing

## Abstract

**Background:**

Pulmonary infections remain a major global health challenge, yet conventional microbiological methods frequently fail to identify causative pathogens. Targeted next-generation sequencing (tNGS) enables rapid, multiplexed detection of bacterial, viral, fungal, and atypical pathogens within a single assay. However, large-scale clinical data validating its diagnostic and epidemiologic value remain limited.

**Methods:**

Pulmonary samples—including sputum, bronchoalveolar lavage fluid (BALF), and throat swabs—were collected from patients with suspected respiratory infections and analyzed using a tNGS assay targeting 153 common pathogens. The diagnostic yield and pathogen spectrum were evaluated, and age-related and clinical-department-specific differences were characterized.

**Results:**

A total of 999 patients were included in this real-world study. tNGS identified pathogens in 844 (84.5%) cases, revealing a diverse microbial landscape with significant age- and season-specific heterogeneity. Viral pathogens predominated in children, whereas bacterial pathogens, including *Mycobacterium tuberculosis*, *Mycoplasma pneumoniae*, and *Pseudomonas aeruginosa*, were more frequent in adults. Mixed infections were common, detected in 70.5% of positive cases.

**Conclusion:**

tNGS enables comprehensive and rapid detection of respiratory pathogens, uncovering complex infection patterns that are often missed by conventional methods. Its application in pulmonary samples, including sputum and BALF, provides valuable diagnostic insights and supports precision management of respiratory infections.

## 1 Introduction

High-throughput sequencing (HTS) technologies have increasingly entered clinical infectious-disease diagnostics, providing broad and sensitive pathogen detection capabilities. Among them, metagenomic next-generation sequencing (mNGS), targeted next-generation sequencing (tNGS), and whole-genome sequencing (WGS) serve distinct but complementary roles. tNGS, in particular, supports high-throughput screening of hundreds of pathogens in a single workflow, enriching target sequences through specific primer amplification or hybrid capture, and enabling detection of both DNA and RNA pathogens at lower cost (Metlay et al., 2019; Jain et al., 2023; World Health Organization, 2024).

Pulmonary infection remains a major global health burden, responsible for substantial morbidity and mortality worldwide (Cilloniz et al., 2022; World Health Organization, 2024). Prompt and accurate pathogen identification is crucial to guide targeted therapy and improve clinical outcomes (Torres et al., 2016; Jain et al., 2023). Current diagnostic workflows rely on clinical assessment—including patient history, symptoms, imaging, and laboratory testing—combined with microbiological assays to identify causative agents. However, conventional approaches are limited by long turnaround times, suboptimal sensitivity, and inability to capture the full spectrum of bacterial, viral, fungal, and atypical pathogens (Wiersinga et al., 2020; Torres et al., 2021; Cilloniz et al., 2022). Consequently, the etiology of pulmonary infections remains undetermined in 19–62% of cases (Torres et al., 2021; Cilloniz et al., 2022).

As a promising technology, tNGS addresses several limitations of mNGS—such as high cost, extensive host-nucleic-acid background, and separate workflows for DNA and RNA detection (Gu et al., 2019; Zhou et al., 2022). Recent studies have demonstrated its clinical utility in respiratory infections. For instance, *Shang* et al (Shang et al., 2025). reported a 97% detection rate using tNGS versus 53% with conventional microbiology in pediatric pneumonia. Similarly, *Yi* et al (Yi et al., 2025). showed that capture-based tNGS achieved 93% diagnostic accuracy and 99% sensitivity, with reduced cost and turnaround time compared with mNGS. In a large clinical evaluation, *Yin* et al (Yin et al., 2024). confirmed that tNGS significantly improved diagnostic yield for lower-respiratory-tract infections when integrated into patient care. A validation study by *Yang* et al (Yang et al., 2025). further demonstrated the robustness of a tNGS workflow for lower-respiratory specimens, while *Jiang* et al (Jiang et al., 2025). emphasized the role of tNGS in overcoming host-background interference in lung infection diagnostics.

Despite these advances, evidence supporting sputum-based tNGS in pulmonary infections remains limited, primarily confined to small case series (Xue et al., 2025). Because sputum sampling is non-invasive, widely acceptable, and more accessible than bronchoalveolar-lavage fluid (BALF), validating tNGS in sputum specimens is of high clinical importance.

Therefore, in this study we applied a tNGS assay targeting 153 pathogens to characterize pathogen-distribution heterogeneity among patients with suspected pulmonary infections and to assess its potential clinical utility in the context of routine conventional microbiological testing.

## 2 Materials and methods

### 2.1 Study design

This retrospective study included 999 consecutive patients with suspected respiratory tract infections who underwent targeted next-generation sequencing (tNGS) testing at Hangzhou First People’s Hospital, Zhejiang, China, between 16 August 2023 and 20 March 2025.

All samples were analyzed using both tNGS and conventional microbiological tests (CMTs), such as smear microscopy, culture, PCR, or serologic testing.

Clinical data—including demographics, radiologic findings, and infection-related laboratory parameters—were extracted from the hospital information system.

The study protocol was approved by the Ethics Committee of Hangzhou First People’s Hospital (approval number: 2022081). All data were anonymized and analyzed solely for research purposes, and strict confidentiality of patient information was maintained. Given the retrospective nature of this study and the use of de-identified data, the requirement for written informed consent was waived by the Ethics Committee of Hangzhou First People’s Hospital.

Inclusion criteria:

- patients diagnosed with pulmonary infection;
- availability of both tNGS and CMT results for pathogen diagnosis;
- complete clinical information;

Exclusion criteria:

- refusal to provide samples for tNGS;
- inadequate sample volume or quality for sequencing; or
- incomplete clinical data.

This was a one-patient, one-sample analysis; only one respiratory specimen per patient was included in the final dataset for tNGS analysis.

### 2.2 Sample collection

Sputum samples were collected following standardized procedures to ensure diagnostic quality.

Trained nurses instructed patients to brush their teeth and rinse with sterile saline in the morning, then take a deep breath and forcefully expectorate sputum from the lower respiratory tract into sterile containers. Contamination with saliva or nasopharyngeal secretions was carefully avoided. For infants or patients unable to expectorate, sputum was collected using a disposable suction tube under negative pressure. When sputum production was difficult, sputum induction or tracheal aspiration was performed by trained clinicians. Approximately 1–3 mL of sputum was collected and stored at −20 °C for up to 48 hours before tNGS analysis. Residual sputum and blood samples from selected patients were simultaneously submitted for CMTs.

### 2.3 Targeted next-generation sequencing analysis

#### 2.3.1 Sample preparation

A 650 μL aliquot of each sample was mixed with an equal volume of 80 mmol/L dithiothreitol in a 1.5 mL centrifuge tube, homogenized for 15 seconds using a vortex mixer, and processed immediately. Positive and negative controls from the KingCreate Respiratory Pathogen Detection Kit (KS608-100HXD96, Guangzhou, China) were included in each batch to monitor assay performance.

#### 2.3.2 Nucleic acid extraction

A 500 μL portion of the homogenate was subjected to total nucleic acid extraction using the MagPure Pathogen DNA/RNA Kit (R6672-01B, Magen, Guangzhou, China) following the manufacturer’s instructions.

#### 2.3.3 Library construction and sequencing

Library preparation was performed using the KingCreate Respiratory Pathogen Detection Kit (KS608-100HXD96), which targets 153 clinically relevant pathogens (bacteria, viruses, fungi, *Mycoplasma*, and *Chlamydia*).

A no-template control was included to monitor library preparation and sequencing.

Libraries underwent two rounds of PCR amplification:

1. Target enrichment using microorganism-specific primer sets for ultra-multiplex amplification, and
2. Adapter/barcode addition for sequencing identification.

Purified PCR products were analyzed using a Qsep100 Bio-Fragment Analyzer (Bioptic, Taiwan) and quantified with a Qubit 4.0 fluorometer (Thermo Fisher Scientific, USA).Typical library fragments ranged from 250–350 bp, with a minimum concentration of 0.5 ng/μL. The pooled libraries were diluted to 1 nmol/L, denatured with 0.1 mol/L NaOH, and sequenced on an Illumina MiniSeq platform using the KingCreate Universal Sequencing Kit (KS107-CXR). Each library generated approximately 0.1 million reads (single-end, 100 bp).

#### 2.3.4 Bioinformatics analysis

Sequencing data were processed using the KingCreate Data Management and Analysis System (v3.7.2). Adaptor sequences and low-quality bases (Q < 20) were removed with Fastp (v0.23). Human reads were filtered out by alignment to the hg38 reference genome using Bowtie2 (v2.3.5). Reads longer than 50 bp and with Q30 > 75% were retained. Filtered reads were aligned against a self-built clinical pathogen reference database, curated from GenBank, RefSeq, and NCBI Nucleotide repositories. Pathogen identification was based on the normalized read count and coverage for each target.

#### 2.3.5 Sub-analysis: mycobacterium tuberculosis complex targeted sequencing

MTBC-targeted sequencing was performed only in a selected subgroup of patients with clinical suspicion of tuberculosis and was not applied uniformly to all 999 study participants. For selected patients with clinical suspicion of tuberculosis, tNGS libraries were prepared using the TB DNA Targeted Library Prep Kit (XP3160-03, Shengshi Zhongfang, Beijing, China).

DNA was extracted with the Sputum Bacterial DNA Kit (XP3170-02, Shengshi Zhongfang), and sequencing was performed on an Illumina NovaSeq 6000 platform (paired-end 150 bp). Sequencing quality was assessed using FastQC, and reads were aligned to the H37Rv reference genome using BWA and Sambamba. Variants were called with GATK, using thresholds of ≥3% allele frequency, ≥5 supporting reads, and ≥1 read in both forward and reverse orientations. Low-frequency resistance or sub-lineage variants were compared against a curated mutation database.

Detection of ≥100 specific sequences across 158 target genes was required to confirm MTBC positivity.

### 2.4 Interpretation of tNGS results

Microbial detection was interpreted in two steps: analytical positivity and clinical adjudication. Analytical positivity was defined using amplicon coverage and normalized read count (reads per million, RPM). For bacteria, fungi, and atypical pathogens (excluding MTBC), positivity required coverage ≥50% and RPM ≥10. For viruses, positivity required either coverage ≥50% with RPM ≥3 or RPM ≥10 when read abundance was high despite incomplete coverage. MTBC positivity was defined as RPM ≥1.

Because respiratory samples may contain colonizing or non-etiologic organisms, analytical positivity alone was not considered sufficient to define causation. Two independent clinicians reviewed all tNGS and CMT results together with the clinical context, including symptoms, chest imaging, inflammatory markers, immune status, and specimen type. A microorganism was classified as clinically relevant only when the detection was concordant with the patient’s presentation and not better explained by colonization, contamination, or an alternative pathogen. In mixed infections, organisms with low pathogenic potential in the respiratory tract, including *Candida albicans* and selected commensal bacteria, were considered etiologic only when supported by compatible host factors and corroborating clinical or microbiological evidence. Discrepancies were resolved by consensus with a senior infectious disease specialist.

### 2.5 Statistical analyses

Statistical analyses were conducted using GraphPad Prism 10 (GraphPad Software, USA). Categorical variables were compared using the χ² or Fisher’s exact test; continuous variables were expressed as medians and compared with the Mann–Whitney U test. A P<0.05 was considered statistically significant.

Subgroup analyses were performed according to age group (≤14 years, 15–59 years, ≥60 years), clinical department (pediatrics, respiratory, infectious disease, ICU, others), and season (spring, summer, autumn, winter).

### 2.6 Ethics statement

The study protocol was reviewed and approved by the Ethics Committee of Hangzhou First People’s Hospital (approval number: 2022081).Because the analysis was based on de-identified retrospective data, the requirement for written informed consent was waived.

## 3 Results:

### 3.1 Overall pathogen detection spectrum by tNGS

#### 3.1.1 Patient characteristics

Between 16 August 2023 and 20 March 2025, a total of 999 patients underwent targeted next-generation sequencing (tNGS) for suspected pulmonary infection in this one-patient, one-sample study. Among these 999 respiratory specimens, 844 (84.5%) were tNGS-positive, whereas 155 (15.5%) yielded no detectable pathogen. Positive specimens included sputum, bronchoalveolar lavage fluid (BALF), and throat swabs. Patient ages ranged from 20 days to 94 years (median = 56 years), comprising 484 males (57.3%) and 360 females (42.7%).Most samples were submitted from the Department of Respiratory Medicine (n = 366), followed by Pediatrics (n = 257), Infectious Diseases (n = 127), and the Intensive Care Unit (n = 47); the remaining 47 samples originated from other clinical departments.

#### 3.1.2 Spectrum of detected pathogens

Across all 844 positive samples, tNGS identified 30 distinct pathogens, comprising 17 viral pathogens, 10 bacterial pathogens (including typical and atypical bacteria), 1 mycobacterial pathogen, and 2 fungal pathogens. The overall detection profile demonstrated marked heterogeneity across age groups and clinical departments.

The most prevalent pathogens were:

- *Mycoplasma pneumoniae* (10.55%),
- *Influenza A virus* (9.00%),
- *Human metapneumovirus* (6.04%),
- *Haemophilus influenzae* (4.98%),
- *Streptococcus pneumoniae* (4.74%), and
- *Pseudomonas aeruginosa* (4.15%).

*Mycobacterium tuberculosis* complex was also detected in 70 cases; however, because MTBC-targeted sequencing was performed only in selected patients with clinical suspicion of tuberculosis, this figure should not be interpreted as the prevalence of MTBC infection in the overall study population.

**Figure 1:**
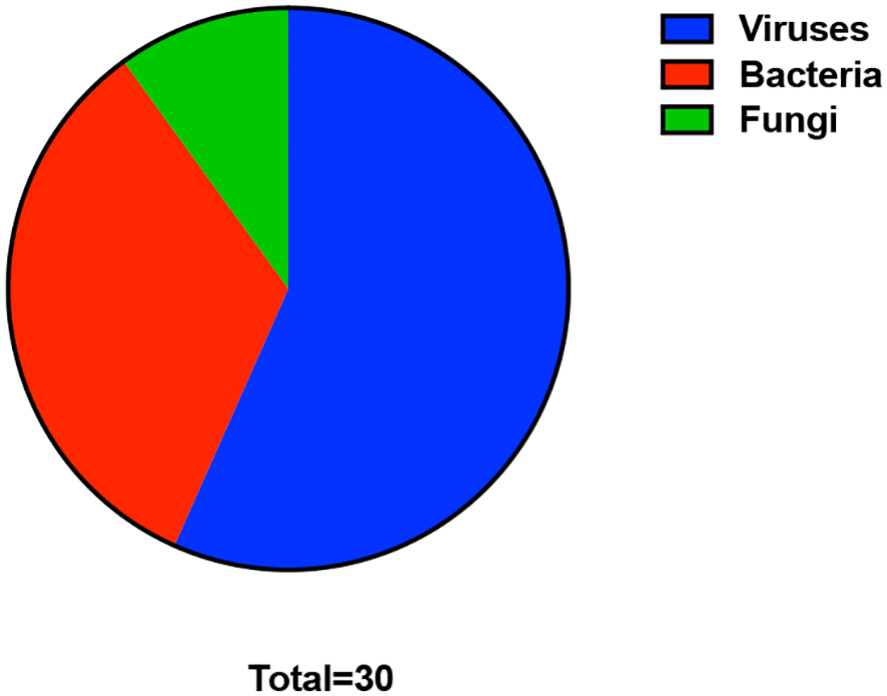
Overall distribution of pathogen categories detected by tNGS.

Fungal pathogens were rarely detected, with only two fungal species identified: *Pneumocystis jirovecii* (1.90%) and *Aspergillus fumigatus* (0.95%). *Mycobacterium tuberculosis* complex was analyzed and reported separately as a mycobacterial pathogen. A detailed breakdown of all 30 pathogens is presented in **Figure 1** and **Table 1**.

**Table 1:** Pathogen detection spectrum among 844 tNGS-positive respiratory samples.

Category	Pathogen	Cases (n)	Proportion (%)
***Viruses (17 total)***	*Influenza A virus*	76	9.00
	*Human metapneumovirus*	51	6.04
	*Respiratory syncytial virus (RSV)*	48	5.69
	*Rhinovirus C*	23	2.73
	*Rhinovirus A*	22	2.61
	*Parainfluenza virus type 3*	18	2.13
	*Human herpesvirus 1 (HSV-1)*	17	2.01
	*Adenovirus species C*	11	1.30
	*Epstein–Barr virus (HHV-4)*	10	1.18
	*Cytomegalovirus (HHV-5)*	10	1.18
	*Coronavirus NL63*	8	0.95
	*SARS-CoV-2*	7	0.83
	*Rhinovirus B*	7	0.83
	*Adenovirus species B*	7	0.83
	*Enterovirus D*	5	0.59
	*Parainfluenza virus type 4*	5	0.59
	*Human herpesvirus 7 (HHV-7)*	5	0.59
***Bacteria, including typical and atypical bacteria (10 total)***	*Mycoplasma pneumoniae*	89	10.55
	*Haemophilus influenzae*	42	4.98
	*Streptococcus pneumoniae*	40	4.74
	*Pseudomonas aeruginosa*	35	4.15
	*Klebsiella pneumoniae*	30	3.55
	*Chlamydia psittaci*	25	2.96
	*Staphylococcus aureus*	18	2.13
	*Chlamydia pneumoniae*	12	1.42
	*Moraxella catarrhalis*	6	0.71
	*Legionella pneumophila*	5	0.59
***Mycobacteria (1 total)***	*Mycobacterium tuberculosis complex*	70	8.29
***Fungi (2 total)***	*Pneumocystis jirovecii*	16	1.90
	*Aspergillus fumigatus*	8	0.95

In summary, the tNGS platform provided a comprehensive overview of the respiratory pathogen landscape in this cohort. Viral pathogens dominated overall detections, while *Mycoplasma pneumoniae* and *Mycobacterium tuberculosis complex* were the leading bacterial and mycobacterial agents, respectively. Fungal infections were comparatively uncommon but clinically relevant in immunocompromised or ICU patients.

Pie chart illustrating the proportion of viruses, bacteria, and fungi identified by tNGS among all positive respiratory samples collected from 999 patients between August 2023 and March 2025.Viral pathogens (blue) accounted for the largest share (56.7%), followed by bacterial pathogens (red, 33.3%) and fungal pathogens (green, 10.0%).

This distribution highlights the predominance of viral infections in the study population and the broad detection capacity of the tNGS platform across major pathogen classes.

Comprehensive list of all pathogens detected by tNGS, categorized as viruses (n = 17), bacteria, including typical and atypical bacteria (n = 10), Mycobacteria (n=1) and fungi (n = 2). For each pathogen, the table shows the number of positive cases and the proportion of total positive samples. The results demonstrate broad detection coverage of the KingCreate KS608 panel, with dominance of viral agents and notable contributions from *Mycoplasma pneumoniae*, *Mycobacterium tuberculosis complex*, and other bacterial pathogens frequently associated with community- and hospital-acquired pneumonia. Note: MTBC was assessed using an additional targeted sequencing workflow in selected patients with clinical suspicion of tuberculosis; therefore, the reported MTBC-positive count should not be interpreted as a prevalence estimate for the entire cohort.

#### 3.1.3 Pathogen distribution according to sample type

**Figure 2:**
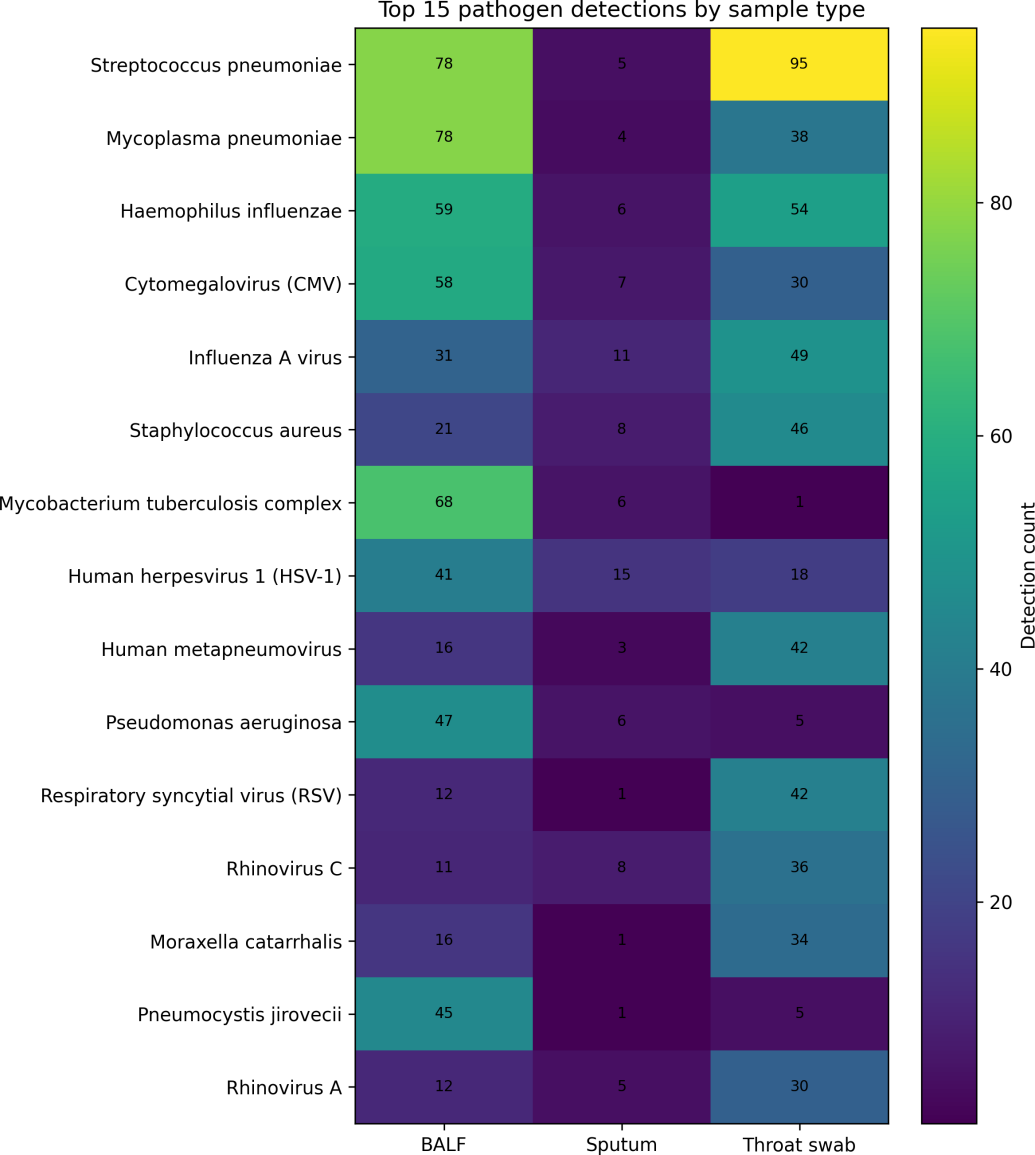
Heatmap of the top 15 pathogen detections by sample type. Heatmap of the top 15 pathogen detections in BALF, throat swab, and sputum specimens. Color intensity corresponds to detection count, and cell labels indicate the exact number of detections. The figure highlights clear differences in pathogen distribution across specimen types.

Pathogen distribution also varied according to specimen type. BALF specimens showed higher frequencies of *Streptococcus pneumoniae*, *Mycoplasma pneumoniae*, *Mycobacterium tuberculosis* complex, and *Haemophilus influenzae*, whereas throat swab specimens more frequently showed *Streptococcus pneumoniae*, Influenza A virus, human metapneumovirus, and respiratory syncytial virus. Sputum specimens showed relatively higher frequencies of HSV-1, *Klebsiella pneumoniae*, and *Acinetobacter baumannii* (**Figure 2**, **Table 2**).

**Table 2:** Major pathogen detections according to sample type.

Rank	BALF	Count	Throat swab	Count	Sputum	Count
1	*Streptococcus pneumoniae*	78	*Streptococcus pneumoniae*	95	Human herpesvirus 1 (HSV-1)	15
2	*Mycoplasma pneumoniae*	78	*Haemophilus influenzae*	54	Influenza A virus	11
3	*Mycobacterium tuberculosis* complex	68	Influenza A virus	49	*Klebsiella pneumoniae*	11
4	*Haemophilus influenzae*	59	*Staphylococcus aureus*	46	*Acinetobacter baumannii*	10
5	Cytomegalovirus (CMV)	58	Human metapneumovirus	42	*Staphylococcus aureus*	8
6	*Pseudomonas aeruginosa*	47	Respiratory syncytial virus (RSV)	42	Rhinovirus C	8
7	*Pneumocystis jirovecii*	45	*Mycoplasma pneumoniae*	38	Human herpesvirus 7	7

This table summarizes the leading pathogen detections identified in bronchoalveolar lavage fluid (BALF), throat swab, and sputum specimens based on the source dataset. Distinct pathogen distributions were observed across specimen types, with BALF enriched for *Streptococcus pneumoniae*, *Mycoplasma pneumoniae*, *Mycobacterium tuberculosis* complex, and *Haemophilus influenzae*; throat swabs showing higher frequencies of *Streptococcus pneumoniae*, Influenza A virus, human metapneumovirus, and respiratory syncytial virus; and sputum showing relatively higher detection of HSV-1, *Klebsiella pneumoniae*, and *Acinetobacter baumannii*.

#### 3.1.4 Comparison between tNGS and conventional microbiological tests

Among the 844 tNGS-positive cases, 334 (39.6%) were also positive by conventional microbiological tests, whereas 510 (60.4%) were negative by CMTs, see **Table 3**. At the pathogen-group level, concordance between tNGS and CMTs varied substantially. For viruses, 67 case-level group occurrences were identified by tNGS only, 287 by CMTs only, and 22 by both methods. For typical bacteria, the corresponding values were 75, 191, and 72; for atypical bacteria, 1, 188, and 76; for mycobacteria, 7, 38, and 41; and for fungi, 57, 10, and 7. The greatest tNGS-only contribution was observed for fungal pathogens, whereas CMT-only group occurrences were more frequent for viruses, typical bacteria, atypical bacteria, and mycobacteria, see **Table 4**.

**Table 3:** Conventional microbiological test results among tNGS-positive cases.

CMT result among tNGS-positive cases	Number of cases	Percentage (%)
CMT positive	334	39.6
CMT negative	510	60.4
Total tNGS-positive cases	**844**	**100.0**

This table summarizes conventional microbiological test (CMT) positivity among the 844 tNGS-positive cases identified in the study cohort. Bold values indicate the total number of tNGS-positive cases and the corresponding overall percentage (100.0%).

**Table 4:** Case-level concordance and discordance between tNGS and conventional microbiological tests by pathogen group.

Pathogen group	Detected by tNGS only, n (%)	Detected by CMT only, n (%)	Detected by both, n (%)	Total grouped detections, n
Viruses	67 (17.8)	287 (76.3)	22 (5.9)	376
Typical bacteria	75 (22.2)	191 (56.5)	72 (21.3)	338
Atypical bacteria	1 (0.4)	188 (70.9)	76 (28.7)	265
Mycobacteria	7 (8.1)	38 (44.2)	41 (47.7)	86
Fungi	57 (77.0)	10 (13.5)	7 (9.5)	74
Total	**207 (18.2)**	**714 (62.7)**	**218 (19.1)**	**1139**

Values represent the number of cases in which at least one pathogen belonging to the indicated group was identified by tNGS only, by CMTs only, or by both methods. Percentages in parentheses were calculated within each pathogen group.

### 3.2 Age-stratified pathogen distribution

#### 3.2.1 Pediatric patients (0–16 years)

Among 265 tNGS-positive pediatric patients, viral pathogens predominated. The five most frequently detected organisms were: *Respiratory syncytial virus* (RSV, 40 cases), *Influenza A virus* (34 cases), *Human metapneumovirus* (34 cases), *Mycoplasma pneumoniae* (32 cases), and *Rhinovirus A* (16 cases).

Distinct age-related trends were evident. RSV and Human metapneumovirus were the dominant pathogens among infants and toddlers (0–4 years), whereas *Mycoplasma pneumoniae* infections rose sharply in school-aged children (>6 years). These findings are consistent with established epidemiologic patterns of viral predominance in early childhood and increasing prevalence of atypical bacterial infections with age, see **Table 5**.

**Table 5:** Age distribution of major pathogens in children (0–16 years, n = 265).

Age Group (years)	RSV	Influenza A	Human Metapneumovirus	*Mycoplasma pneumoniae*	Rhinovirus A	Total detections (n)
0–1	15	2	4	0	3	24
1–2	6	7	3	0	1	17
2–3	6	3	5	1	3	18
3–4	7	4	9	0	4	24
4–5	2	9	3	2	2	18
5–6	0	1	4	2	1	8
6–8	4	4	4	13	1	26
8–10	0	2	1	5	1	9
10–12	0	1	0	5	0	6
12–14	0	1	1	1	0	3
14–16	0	0	0	3	0	3
Total Cases	**40**	**34**	**34**	**32**	**16**	**156**

The bold values indicate the total number of cases and the corresponding overall percentage within each table section, presented for emphasis as summary values.

This table summarizes the age-specific detection frequencies of the five predominant pathogens identified by targeted next-generation sequencing (tNGS) among pediatric patients. Viral agents—particularly *Respiratory syncytial virus* (RSV), *Influenza A virus*, and *Human metapneumovirus*—were most common in infants and toddlers (0–4 years), whereas *Mycoplasma pneumoniae* infections increased markedly in school-aged children. *Rhinovirus A* was detected across multiple age brackets with lower frequency. These results illustrate the characteristic shift from viral to atypical bacterial dominance with increasing age.

#### 3.2.2 Adult patients (16–94 years)

**Figure 3:**
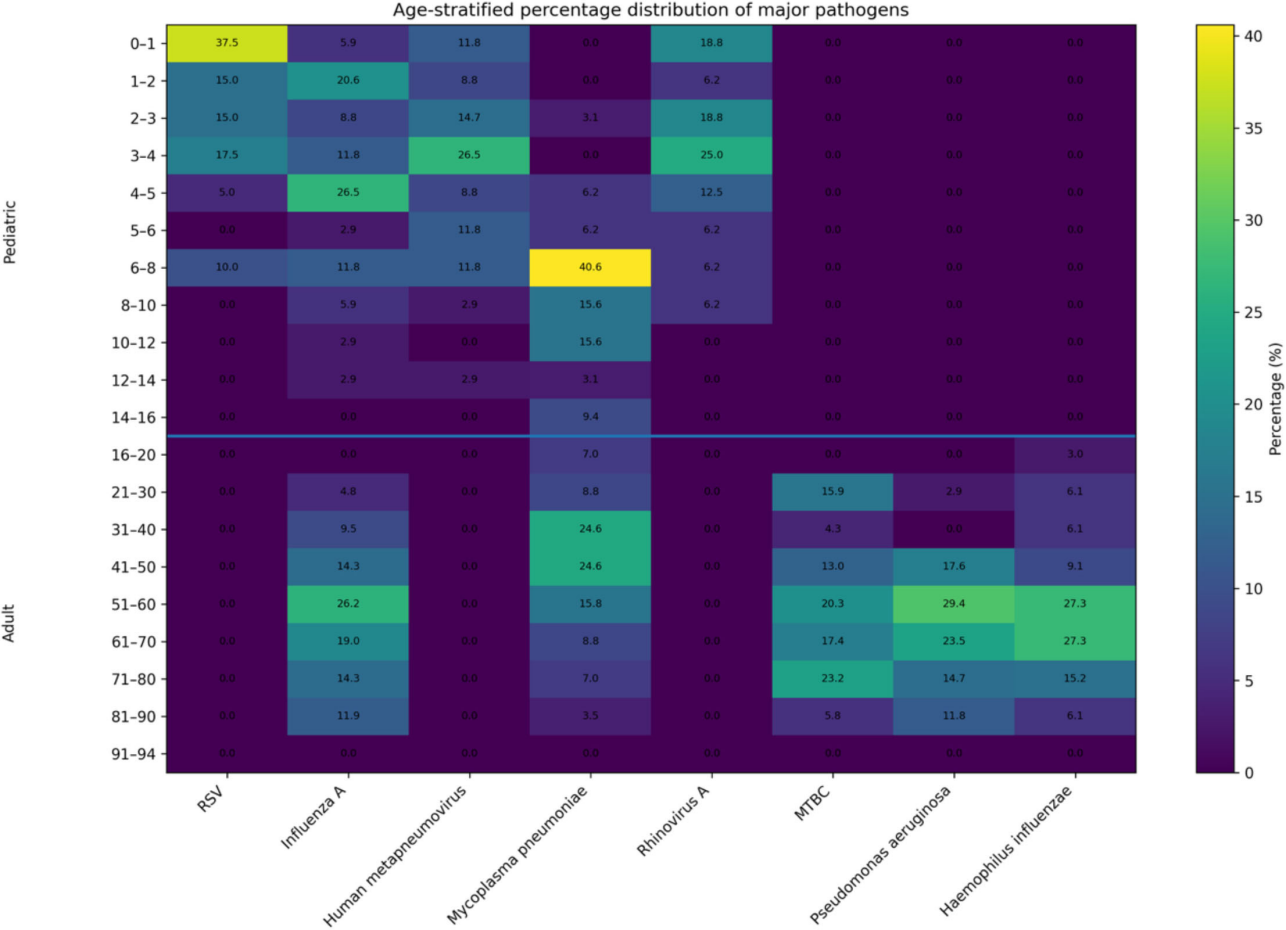
Heatmap of age-stratified distribution of major respiratory pathogens detected by tNGS.

Among 579 tNGS-positive adults, bacterial and mycobacterial pathogens were more prevalent than viral agents. The leading organisms included *Mycobacterium tuberculosis complex* (69 cases), *Mycoplasma pneumoniae* (57 cases), *Influenza A virus* (42 cases), *Pseudomonas aeruginosa* (34 cases), and *Haemophilus influenzae* (33 cases). *Mycobacterium tuberculosis* showed a relatively uniform distribution across adult age groups, while *M. pneumoniae*, *H. influenzae*, and *P. aeruginosa* were more frequently detected among middle-aged and elderly patients (41–80 years),see **Figure 3** and **Table 6**.

**Table 6:** Age distribution of major pathogens in adults (16–94 years, n = 579).

Age group (years)	*Mycobacterium tuberculosis*	*Mycoplasma pneumoniae*	Influenza A virus	*Pseudomonas aeruginosa*	*Haemophilus influenzae*	Total detections (n)
16–20	0	4	0	0	1	**5**
21–30	11	5	2	1	2	**21**
31–40	3	14	4	0	2	**23**
41–50	9	14	6	6	3	**38**
51–60	14	9	11	10	9	**53**
61–70	12	5	8	8	9	**42**
71–80	16	4	6	5	5	**36**
81–90	4	2	5	4	2	**17**
91–94	0	0	0	0	0	**0**
Total Cases	**69**	**57**	**42**	**34**	**33**	**235**

The bold values indicate the total number of cases and the corresponding overall percentage within each table section, presented for emphasis as summary values.

These results indicate that chronic airway disease, immune senescence, and comorbidities may contribute to pathogen variability across the adult lifespan.

This table presents the distribution of the five most frequently detected pathogens among adult patients with tNGS-positive pulmonary infections. *Mycobacterium tuberculosis complex* remained consistently prevalent across all age groups, while *Mycoplasma pneumoniae*, *Haemophilus influenzae*, and *Pseudomonas aeruginosa* were more common in middle-aged and elderly individuals. *Influenza A virus* showed seasonal clustering but was also detected broadly throughout adulthood. The data underscore the predominance of bacterial and mycobacterial infections in adults compared with the viral predominance seen in children.

Heatmap illustrating the percentage distribution of the five most common pathogens across age groups in both pediatric (0–16 years) and adult (16–94 years) patients. Each cell represents the percentage of detections for a given pathogen occurring within the corresponding age group, with color intensity reflecting the relative proportion. Viral pathogens, including respiratory syncytial virus (RSV), Influenza A virus, and human metapneumovirus, were concentrated in younger children, whereas *Mycoplasma pneumoniae* increased in school-aged patients. In adults, *Mycobacterium tuberculosis* complex, *Haemophilus influenzae*, and *Pseudomonas aeruginosa* were more frequently distributed among middle-aged and elderly groups.

### 3.3 Department-specific pathogen patterns

Marked differences in pathogen distribution were observed across the four major clinical departments.

Respiratory Medicine: *Mycobacterium tuberculosis complex*, *Mycoplasma pneumoniae*, *Haemophilus influenzae*, and *Pseudomonas aeruginosa* were most frequent.

Pediatrics: Infections were dominated by viral pathogens—mainly *Respiratory syncytial virus* (RSV), *Influenza A virus*, *Human metapneumovirus*, *Mycoplasma pneumoniae*, and rhinoviruses (A and C).

Infectious Diseases: *Mycobacterium tuberculosis* was the leading organism, followed by *Influenza A virus*, *Mycoplasma pneumoniae*, *Chlamydia psittaci*, and *Streptococcus pneumoniae*.

Intensive Care Unit (ICU): Severe bacterial infections predominated, with high detection rates of *Klebsiella pneumoniae*, *Pseudomonas aeruginosa*, *Acinetobacter baumannii*, and *Enterobacter cloacae*.

**Figure 4:**
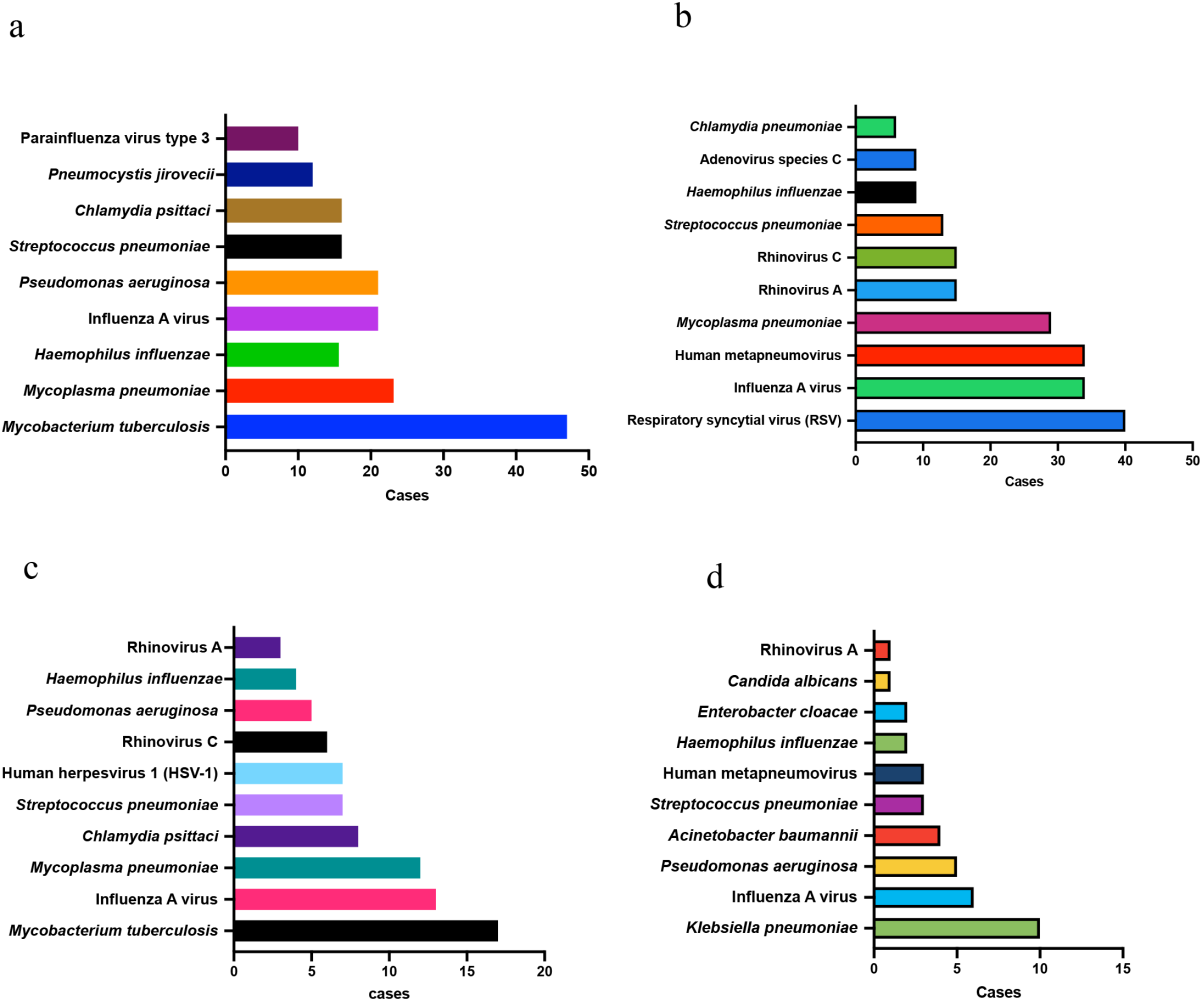
Department-specific distribution of major respiratory pathogens detected by tNGS. (**a**) Respiratory Medicine, **(b)** Pediatrics, **(c)** Infectious Diseases, and **(d)** Intensive Care Unit (ICU) departments.

These departmental variations likely reflect underlying patient conditions, immune status, and infection severity, emphasizing the need to interpret tNGS results within the clinical context, see **Figure 4** and **Table 7**.

**Table 7:** Top 10 pathogens detected by tNGS across four major clinical departments.

Department	Rank	Pathogen	Cases (n)	Proportion of department (%)
Respiratory Medicine (n = 366)	1	*Mycobacterium tuberculosis*	47	**48.63%** (178/366 total positives)
	2	*Mycoplasma pneumoniae*	44	
	3	*Haemophilus influenzae*	27	
	4	Influenza A virus	21	
	5	*Pseudomonas aeruginosa*	21	
	6	*Streptococcus pneumoniae*	16	
	7	*Chlamydia psittaci*	16	
	8	*Pneumocystis jirovecii*	12	
	9	*Klebsiella pneumoniae*	11	
	10	Parainfluenza virus type 3	10	
				
Pediatrics (n = 257)	1	Respiratory syncytial virus (RSV)	40	**63.81%** (164/257 total positives)
	2	*Influenza A virus*	34	
	3	*Human metapneumovirus*	34	
	4	*Mycoplasma pneumoniae*	29	
	5	*Rhinovirus A*	15	
	6	*Rhinovirus C*	15	
	7	*Streptococcus pneumoniae*	13	
	8	*Haemophilus influenzae*	9	
	9	Adenovirus species C	9	
	10	*Chlamydia pneumoniae*	6	
				
Infectious Diseases (n = 127)	1	*Mycobacterium tuberculosis*	17	**51.18%** (65/127 total positives)
	2	*Influenza A virus*	13	
	3	*Mycoplasma pneumoniae*	12	
	4	*Chlamydia psittaci*	8	
	5	*Streptococcus pneumoniae*	7	
	6	*Human herpesvirus 1 (HSV-1)*	7	
	7	*Rhinovirus C*	6	
	8	*Pseudomonas aeruginosa*	5	
	9	*Haemophilus influenzae*	4	
	10	Rhinovirus A	3	
				
ICU (n = 47)	1	*Klebsiella pneumoniae*	10	**57.45%** (27/47 total positives)
	2	*Influenza A virus*	6	
	3	*Pseudomonas aeruginosa*	5	
	4	*Acinetobacter baumannii*	4	
	5	*Streptococcus pneumoniae*	3	
	6	*Human metapneumovirus*	3	
	7	*Haemophilus influenzae*	2	
	8	*Enterobacter cloacae*	2	
	9	*Candida albicans*	1	
	10	Rhinovirus A	1	

The bold values indicate the total number of cases and the corresponding overall percentage within each table section, presented for emphasis as summary values.

This table summarizes the ten most frequently detected pathogens in each department.

In Respiratory Medicine, *Mycobacterium tuberculosis* and *Mycoplasma pneumoniae* were predominant; Pediatrics was dominated by viral pathogens such as RSV and *Influenza A virus*; Infectious Diseases showed a mixture of *Mycobacterium tuberculosis* and atypical bacteria; and ICU samples demonstrated a higher burden of multidrug-resistant Gram-negative bacteria. These patterns illustrate the influence of patient population and disease severity on pathogen composition.

Each horizontal bar represents the number of cases attributed to the top ten pathogens detected by targeted next-generation sequencing (tNGS) in that department.

Distinct pathogen profiles were observed across clinical settings: (a) Respiratory Medicine showed high prevalence of *Mycobacterium tuberculosis complex*, *Mycoplasma pneumoniae*, *Haemophilus influenzae*, and *Pseudomonas aeruginosa*. (b) Pediatrics was dominated by viral infections, particularly *Respiratory syncytial virus* (RSV), *Influenza A virus*, and *Human metapneumovirus*. (c) Infectious Diseases exhibited mixed patterns led by *Mycobacterium tuberculosis* and atypical bacteria such as *Chlamydia psittaci*. (d) ICU samples revealed enrichment of multidrug-resistant Gram-negative bacteria, including *Klebsiella pneumoniae*, *Pseudomonas aeruginosa*, and *Acinetobacter baumannii*. The figure highlights the marked heterogeneity in pathogen distribution across departments, reflecting differences in patient populations, immune status, and infection severity.

### 3.4 Mixed vs. single infections

**Figure 5:**
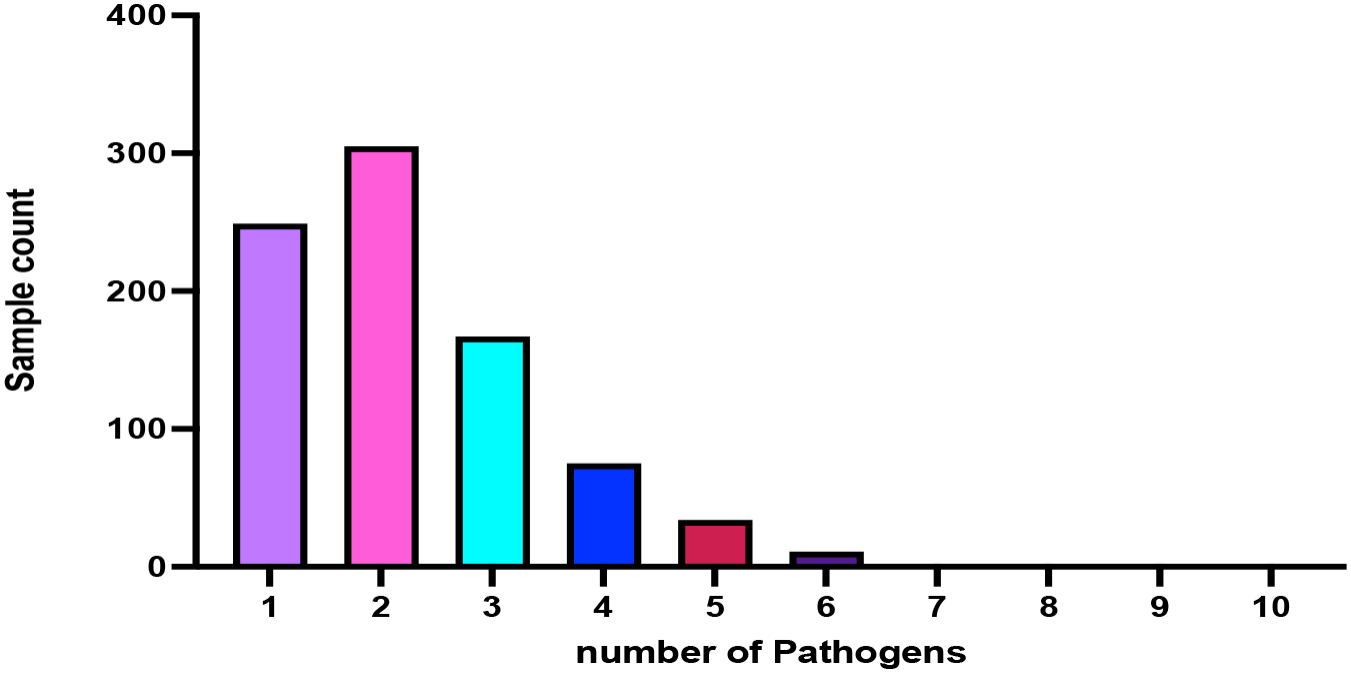
Distribution of single and mixed infections detected by tNGS.

Mixed infections were common in the study cohort. Among 844 tNGS-positive samples, 595 cases (70.5%) involved detection of more than one pathogen, whereas 249 cases (29.5%) contained a single pathogen. Two-pathogen co-infections were the most frequent pattern (305/844, 36.14%), followed by three-pathogen (167/844, 19.78%), four-pathogen (75/844, 8.89%), five-pathogen (34/844, 4.03%), and six-pathogen (11/844, 1.66%) detections. These data indicate that polymicrobial detection was a prominent feature of respiratory specimens analyzed by tNGS in this cohort, see **Figure 5** and **Table 8**.

**Table 8:** Distribution of single and mixed infections detected by tNGS (n = 844 positive cases).

Number of pathogens detected per sample	Sample count (n)	Proportion (%)
Single-pathogen infections	249/844	29.50
Mixed infections (≥2 pathogens)	595/844	70.5
Two-pathogen co-infections	305/844	36.14
Three-pathogen co-infections	167/844	19.78
Four-pathogen co-infections	75/844	8.89
Five-pathogen co-infections	34/844	4.03
Six-pathogen co-infections	11/844	1.66
Seven pathogens	0	
Eight pathogens	2/844	
Nine pathogens	0	
Ten pathogens	1/844	
Total	**844**	**100%**

The bold values indicate the total number of cases and the corresponding overall percentage within each table section, presented for emphasis as summary values.

Viral–viral and viral–bacterial combinations were predominant, whereas fungal co-infections occurred mainly in immunocompromised or ICU patients, see **Table 9**. These findings underscore the clinical importance of comprehensive molecular diagnostics for accurate infection profiling and targeted antimicrobial therapy.

**Table 9:** Illustrative composition of mixed infections.

Co-infection class	Representative pathogen label	Illustrative count out of 595
Viral–viral mixed infections	Adenovirus spp. + Rhinovirus spp.	**68**
Viral–bacterial mixed infections	Rhinovirus spp. + Haemophilus influenzae	**286**
Bacterial–bacterial mixed infections	Haemophilus influenzae + Streptococcus pneumoniae	**160**
Other/complex mixed infections	Higher-order mixed combinations (≥3 pathogens)	**81**
Total		**595**

Schematic distribution of the 595 mixed infections detected in the present cohort, shown as viral–viral, viral–bacterial, bacterial–bacterial, and other/complex categories.

The bold values indicate the total number of cases and the corresponding overall percentage within each table section, presented for emphasis as summary values.

Bar chart showing the proportion of samples harboring one or multiple pathogens among 844 tNGS-positive respiratory specimens. Mixed infections accounted for 70.5% of positive cases, with two-pathogen co-infections being most prevalent, followed by three- and four-pathogen combinations. Fungal co-infections (≤2%) were mainly observed in ICU or immunocompromised patients. This figure highlights the predominance of mixed microbial infections in respiratory disease and the comprehensive detection ability of targeted next-generation sequencing.

This table summarizes the number of pathogens identified per sample among tNGS-positive respiratory specimens. Single infections accounted for 29.5% of positive samples, while mixed infections constituted 70.5%, with dual-pathogen infections being most common. The data highlight tNGS’s capability to uncover multi-pathogen co-infections that may be under-recognized by conventional diagnostic methods.

### 3.5 Seasonal distribution of infections (2024)

**Figure 6:**
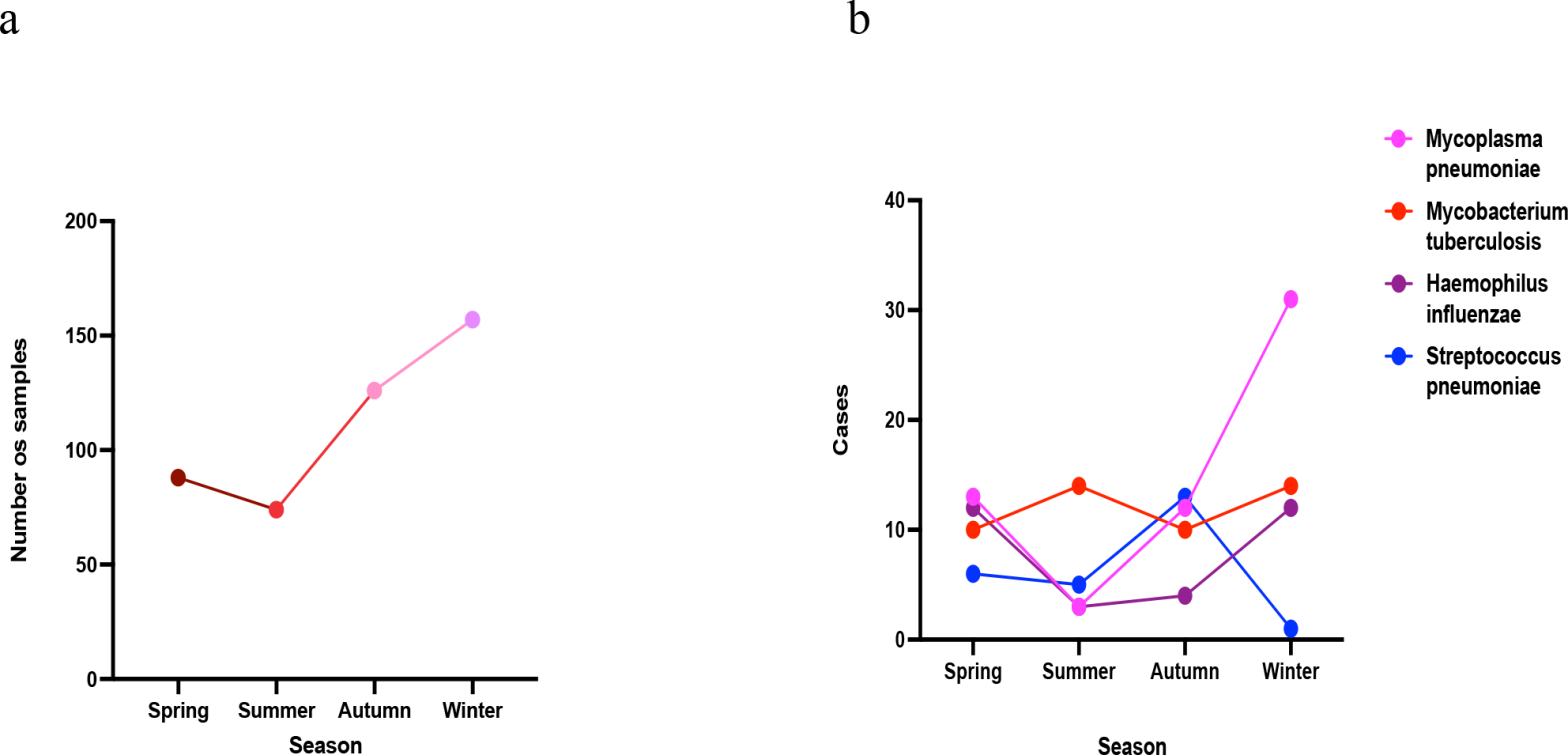
Seasonal trends of tNGS-positive respiratory infections in 2024. (**a**) Seasonal distribution of overall tNGS-positive cases. Line graph showing the total number of tNGS-positive respiratory samples detected across four seasons (spring, summer, autumn, and winter). A pronounced peak was observed in winter (35.3%), followed by autumn (28.3%), reflecting heightened respiratory infection activity during colder months. **(b)** Seasonal variation of the four most common pathogens.

A total of 445 tNGS-positive cases in 2024 displayed clear seasonal trends: Winter: 157 cases (35.3%), Autumn: 126 cases (28.3%), Spring: 88 cases (19.8%), Summer: 74 cases (16.6%). The four most common pathogens exhibited distinct seasonality: *Mycoplasma pneumoniae* peaked in autumn → winter → spring, with a marked drop in summer. *Mycobacterium tuberculosis* remained relatively constant year-round, consistent with known epidemiology. *Haemophilus influenzae* predominated in winter and spring. *Streptococcus pneumoniae* occurred mainly in autumn, see **Table 10** and **Figure 6**. These seasonal differences align with known respiratory pathogen epidemiology and climatic influences.

**Table 10:** Seasonal distribution of the four most common pathogens in 2024.

Pathogen	Spring(n=88)	Summer(n=74)	Autumn(n=126)	Winter(n=157)	Total (n)
Mycoplasma pneumoniae	13(14.8%)	3(4.1%)	12(9.5%)	31(19.7%)	59
Mycobacterium tuberculosis	10(11.4%)	14(18.9%)	10(7.9%)	14(8.9%)	48
Haemophilus influenzae	12(13.6%)	3(4.1%)	4(3.2%)	12(7.6%)	31
Streptococcus pneumoniae	6(6.8%)	5(6.8%)	13(10.3%)	1(0.6%)	25
Subtotal number of detection	41(46.6%)	25(33.8%)	39(31%)	58(36.9%)	163

This table details the seasonal variation in detection frequencies of the four leading pathogens among tNGS-positive samples. *Mycoplasma pneumoniae* showed the strongest seasonal fluctuation with a peak in winter, whereas *Mycobacterium tuberculosis* remained steady across seasons. *Haemophilus influenzae* was most frequent in winter and spring, and *Streptococcus pneumoniae* predominated in autumn.

Line graph illustrating the seasonal detection frequencies of *Mycoplasma pneumoniae*, *Mycobacterium tuberculosis*, *Haemophilus influenzae*, and *Streptococcus pneumoniae*. *Mycoplasma pneumoniae* demonstrated a marked winter peak, *Mycobacterium tuberculosis* remained relatively stable year-round, *Haemophilus influenzae* was most common in winter and spring, and *Streptococcus pneumoniae* peaked in autumn. Together, these patterns align with known epidemiologic trends of respiratory infections in temperate climates.

### 4 Discussion

This large real-world study demonstrates the clinical utility of targeted next-generation sequencing (tNGS) for pathogen identification in pulmonary infections. Across 999 samples, tNGS revealed a rich and heterogeneous pathogen spectrum, highlighted distinct age-specific and seasonal trends, and uncovered a high prevalence of mixed infections. These findings reinforce the diagnostic advantages of tNGS and provide new insights into the epidemiology of respiratory infections in a diverse clinical population.

Although tNGS showed a substantially higher overall case-level positivity rate in the comparison subset, pathogen-group concordance varied across organism classes, suggesting that tNGS and CMTs may provide complementary rather than uniformly interchangeable diagnostic information.

### 4.1 Heterogeneous pathogen spectrum revealed by tNGS

The pathogen distribution identified by tNGS differed substantially from patterns reported by conventional microbiological tests. Viral pathogens dominated pediatric infections, whereas bacterial pathogens—including *Mycobacterium tuberculosis*, *Mycoplasma pneumoniae*, and *Pseudomonas aeruginosa*—were more common in adults. Mycobacterium tuberculosis complex was frequently detected in our dataset, particularly among adult and respiratory/infectious disease subgroups; however, because MTBC-targeted sequencing was performed only in selected clinically suspected cases, the observed MTBC-positive count should be interpreted cautiously and not as the true prevalence of tuberculosis in the whole cohort. The ability of tNGS to detect viruses, atypical bacteria, and multiple co-infecting organisms in a single assay likely explains its broader detection spectrum relative to routine culture or PCR (Zhou et al., 2021; Guo et al., 2022). The relatively low detection rate of fungal pathogens reflects their lower prevalence in the general population and the fact that invasive fungal infections primarily occur in immunocompromised hosts (Arastehfar et al., 2021).

### 4.2 Age-related differences in infection patterns

Children exhibited a pathogen profile dominated by RSV, influenza viruses, *Human metapneumovirus*, and rhinoviruses—consistent with established epidemiology, where viral respiratory infections are more prevalent in younger age groups due to immature immunity and high exposure in community settings (Li et al., 2022; Charlton et al., 2019). In contrast, adults—especially older adults—showed a higher burden of *Mycobacterium tuberculosis*, *Pseudomonas aeruginosa*, *Klebsiella pneumoniae*, and *Haemophilus influenzae*, consistent with the effects of chronic lung disease, comorbidities, and prior antibiotic exposure (Rudan et al., 2022; Cilloniz and Torres, 2023).

### 4.3 Virulence, infection complexity, and pathogen interactions

Our cohort suggests substantial variation in infection complexity across detected pathogens. Highly virulent organisms—such as *Mycobacterium tuberculosis*, *Pseudomonas aeruginosa*, and *Streptococcus pneumoniae*—were more often detected as single-pathogen infections, suggesting that intrinsic pathogenicity enables these organisms to dominate the respiratory microenvironment and suppress competitors. Conversely, low-virulence pathogens, including many respiratory viruses, appeared more frequently in mixed infections, likely reflecting differences in viral load, competitive fitness, and virulence-gene expression (Kiedrowski and Bomberger, 2023; Lima et al., 2023). however, formal analysis of virulence categories and co-infection burden was beyond the scope of the present study. These observations should therefore be interpreted as hypothesis-generating.

The frequent viral–viral and viral–bacterial co-detections observed in this study suggest potential synergistic or antagonistic interactions among respiratory pathogens. Previous reports indicate that viral co-infections can predispose hosts to secondary bacterial pneumonia or, conversely, interfere with other viral replication through competitive inhibition (Bosch et al., 2013; Nickbakhsh et al., 2019). These findings highlight the complex microbial ecology of the respiratory tract and underscore tNGS’s diagnostic advantage in capturing these interactions.

### 4.4 High prevalence and clinical implications of mixed infections

Mixed infections accounted for more than 70% of positive cases. Two-pathogen combinations were the most frequent, though several samples harbored up to six pathogens. This high co-infection rate likely reflects both the enhanced sensitivity of tNGS and the dynamic microbial interactions within the airway ecosystem (Simner et al., 2022; Zhang et al., 2023). These results emphasize that clinicians should interpret tNGS findings within the context of potential polymicrobial synergy and host immune status when tailoring antimicrobial therapy.

### 4.5 Seasonal variations in respiratory pathogens

Consistent with known respiratory pathogen behavior, *Mycoplasma pneumoniae* demonstrated cyclical peaks in autumn and winter, while influenza and RSV clustered strongly in colder months (Pan et al., 2024; Cao et al., 2024). *Mycobacterium tuberculosis* maintained a relatively stable pattern year-round, consistent with its chronic infection dynamics (Taylan et al., 2023). These seasonal findings are in line with recent national surveillance data confirming winter dominance of viral infections and autumn peaks of *M. pneumoniae* (Chinese Center for Disease Control and Prevention, 2025).

### 4.6 Department-specific pathogen patterns

Department-specific pathogen profiles reflected patient demographics and disease severity. ICU patients exhibited higher frequencies of multidrug-resistant Gram-negative bacteria—*Klebsiella pneumoniae*, *Pseudomonas aeruginosa*, and *Acinetobacter baumannii*—commonly implicated in hospital-acquired pneumonia (Ramachandran et al., 2024). Pediatric cases showed dominance of classic viral agents, whereas Respiratory Medicine and Infectious-Disease departments demonstrated higher burdens of tuberculosis and atypical bacteria, consistent with prior studies (Chen et al., 2018).

### 4.7 Strengths of tNGS in pulmonary infection diagnosis

tNGS offers several advantages:

- Broad pathogen coverage spanning viruses, bacteria, atypical organisms, and fungi.
- Ability to detect mixed infections in a single workflow.
- Shorter turnaround compared with culture.
- Improved sensitivity for fastidious or non-culturable pathogens.
- Simultaneous DNA/RNA detection.
- Lower cost than mNGS, facilitating clinical scalability (Li et al., 2022; Sun et al., 2024; Yin et al., 2024; Shang et al., 2025; Yi et al., 2025).

Collectively, these strengths position tNGS as a robust complement to conventional microbiological testing and a promising approach for improved diagnostic precision.

### 4.8 Limitations

Several limitations should be acknowledged. First, this retrospective single-center design may limit generalizability. Second, clinical outcome data were not analyzed alongside tNGS results, precluding direct assessment of therapeutic impact. Third, quantitative microbial load was not determined, limiting inference on pathogen dominance in mixed infections. Finally, some organisms may represent colonization rather than infection. Future multicenter prospective studies with outcome integration and standardized interpretation thresholds are warranted (Chen et al., 2024).

## 5 Conclusions

This study demonstrates the real-world clinical value of targeted next-generation sequencing in characterizing the pathogen landscape of pulmonary infections. tNGS enabled broad pathogen detection, revealed significant age- and season-specific trends, and identified a high burden of mixed infections. These results underscore tNGS’s potential to enhance diagnostic accuracy, support antimicrobial stewardship, and inform precision treatment strategies in respiratory infectious disease management.

## Data Availability

The datasets generated in this study derive from sequencing of human clinical respiratory samples and may contain potentially identifiable human sequence information. Therefore, the raw data are not currently publicly available pending confirmation of the applicable ethics and institutional data-sharing requirements. Requests for access to de-identified data should be directed to the corresponding author. If repository deposition is approved, accession number(s) and direct link(s) will be provided prior to publication.
